# A computational approach for intramural length estimation in anomalous aortic origin of a coronary artery

**DOI:** 10.3389/fcvm.2025.1721523

**Published:** 2026-02-02

**Authors:** Vikram Shah, Lauren Ferrino, Dana Reaves-O’Neal, Tam T. Doan, Shagun Sachdeva, Craig G. Rusin, Dan Lior, Charles Puelz, Prakash M. Masand, Silvana Molossi

**Affiliations:** 1 Department of Computational Applied Mathematics and Operations Research, Rice University, Houston, TX, United States; 2 Department of Pediatrics, Division of Cardiology, Baylor College of Medicine and Texas Children’s Hospital, Houston, TX, United States; 3 Department of Medicine, Baylor College of Medicine, Houston, TX, United States; 4 Department of Mathematics, University of Houston, Houston, TX, United States; 5 Edward B. Singleton Department of Radiology, Baylor College of Medicine and Texas Children’s Hospital, Houston, TX, United States

**Keywords:** anomalous aortic origin of a coronary artery, computed tomography angiography, intramural length, risk stratification, sudden cardiac arrest

## Abstract

**Introduction:**

Anomalous aortic origin of a coronary artery (AAOCA) is associated with sudden cardiac death. The intramural (IM) length is considered high-risk, yet radiologic measurements by computed tomography angiography (CTA) show variable agreement with measurements at surgery. We aimed to develop a semi-automatic computational method to estimate IM length in a retrospective cohort of surgical AAOCA patients.

**Methods:**

In 58 patients [49 right(R), 9 left(L)], CTA images were used to generate 3D segmentations of the aorta and a centerline of the anomalous coronary. The distance from the centerline to the aortic segmentation was calculated. The IM length was estimated from a transition point in the derivative of the distance curve and compared to radiologic and surgical measurements.

**Results:**

Our method demonstrated an overall root-mean-square error (RMSE) of 3.4 mm, comparable to radiologic estimates (3.2 mm). For L-AAOCA subjects, our method showed lower root-mean-square error compared to radiologic estimates (our method: 3.6 mm, radiologic: 4.7 mm). For R-AAOCA subjects, the RMSE was higher in our method compared to radiologic estimates (our method: 3.4 mm, radiologic: 2.8 mm).

**Conclusion:**

This is a pilot study of a computational approach to measure intramural length that is shown to be accurate relative to surgical measurements. Computational methods that represent and quantify morphology, including acute take-off angle, ostial characteristics, minimal luminal area, and intramural length, may be helpful for risk stratification and surgical planning in AAOCA.

## Introduction

Anomalous aortic origin of a coronary artery (AAOCA) is associated with increased risk of myocardial ischemia and sudden cardiac death in the young (1–7). Enhanced risk assessment is needed to improve clinical management in pediatric patients (8). Current practice for risk prediction relies on coronary computed tomography angiography (CTA), exercise stress testing, and stress perfusion imaging.

**Table 1:** Cohort of patients including type of anomaly (anomalous origin of the left or right coronary artery), sinus of origin (1 or 2), location within the sinus (a–c), and level in the aorta (I-IV) on CTA images (17).

Patient	Anomaly	Ostium Location	Intramural Length Estimates (mm)			Surgical Repair (R = Reimplantation, U = Unroofing)
			Radiologic	Computational	Surgical	
1	L-AAOCA	1c, IV	0	4.2	6	U
2	L-AAOCA	1c, III/IV	3–4	4.9	1–2	U
3	L-AAOCA	2c, II	2.8	8.3	0	R
4	L-AAOCA	Unknown	10	6.4	7	U
5	L-AAOCA	1c, II	3	8.6	8	R
6	L-AAOCA	1c, II	8.9	4.3	9	U
7	L-AAOCA	1b, II	0 (or “Short”)	8.6	10	U
8	L-AAOCA	1c, II	0	3.7	4	U
9	L-AAOCA	1c, II/III	10	13.9	11	U
10	R-AAOCA	2a, II/III	6–7	3.9	5–6	U
11	R-AAOCA	2a, III	2.5	3.3	3	U
12	R-AAOCA	2a, III	8.2	3.7	7	U
13	R-AAOCA	2a, II	4.8–5	9.4	4.5	U
14	R-AAOCA	2a, I	8.5–9	2.9	6	U
15	R-AAOCA	2a, II	6.8	7.9	6	U
16	R-AAOCA	2a, II	4–4.4	9	4	U
17	R-AAOCA	2a, III	7.4	8.5	7	U
18	R-AAOCA	2a, II	12	4.2	4	U
19	R-AAOCA	2a, II	8.8–10.8	11.2	4	U
20	R-AAOCA	2a, III	12	9.6	9	U
21	R-AAOCA	2a, III	4	6.4	4	R
22	R-AAOCA	2a, III	6	5.9	6	U
23	R-AAOCA	2a, III	6	7.2	6	R
24	R-AAOCA	2a, II	2.4	7.5	3	U
25	R-AAOCA	2a, II	9–9.5	3.8	9	U
26	R-AAOCA	2a, II/III	8.5	1.5	0 (or “Short”)	R
27	R-AAOCA	Unknown	3.5	9.3	7	U
28	R-AAOCA	2a, II	5.8	10	6	U
29	R-AAOCA	Unknown, III	6	4.3	5	U
30	R-AAOCA	2b, IV	6.5–7	8.1	6.5	U
31	R-AAOCA	2a, IV	5–6	6.9	5	U
32	R-AAOCA	2a, II	3	1.5	5	U
33	R-AAOCA	2a, I	6	11.4	8	R
34	R-AAOCA	2a, II	8	11.1	7	U
35	R-AAOCA	2a, III	7	4.3	9	U
36	R-AAOCA	2a, III	7	5.7	6	U
37	R-AAOCA	2a, II	11–12	6.8	8	U
38	R-AAOCA	2a, III	9	6.3	5	U
39	R-AAOCA	2a, II	7.4	6	6	U
40	R-AAOCA	2a, III	6	6.6	6	U
41	R-AAOCA	2c, IV	4	0.4	4	U
42	R-AAOCA	2a, III	3–4	8.4	3	U
43	R-AAOCA	Unknown, IV	4.4	5.3	5	U
44	R-AAOCA	2a, II	7	7.1	3	U
45	R-AAOCA	2a, II	8	10	7	U
46	R-AAOCA	2a, II	9.5	6.5	7	U
47	R-AAOCA	2a, II	9–9.4	1.3	8	U
48	R-AAOCA	2a, II	6.5	6.8	6	U
49	R-AAOCA	2a, II	5.8	9.9	5	R
50	R-AAOCA	2a, II	4	6.2	4	R
51	R-AAOCA	2a, II	8	4	5	U
52	R-AAOCA	2a, II	8.5–10	4.2	7	U
53	R-AAOCA	2a, II	7.6	2.9	7	R
54	R-AAOCA	2a, III	6–7	23.1	16	R
55	R-AAOCA	2a, I	6	10.1	6	R
56	R-AAOCA	2a, II	7	12	8	U
57	R-AAOCA	2a, II	9	9.6	9	U
58	R-AAOCA/LAD MB	Unknown	7	4	10	U

The cases of L-AAOCA had an interarterial course between the aorta and pulmonary trunk. The radiologic estimate from the CTA, computational estimate by our method, and the intraoperative surgical measurements are listed. The ostium location was not defined for specific characteristics in five patients (“Unknown”). Patient 58 had anomalous aortic origin of the right coronary artery associated with a myocardial bridge of the left anterior descending coronary artery. If the radiologic estimate or surgical measurement was reported as a range of values in the clinical report, the average was used for error calculations (for example, patient 2 had a range of 3–4 mm for their radiologic estimate, so 3.5 mm was used for the radiologic IM length). We assumed that anything less than 3 mm would be reasonable to label as a “short” IM length in this context. In cases where the radiologic or surgical IM length values were reported as “short” on clinical reports, 0 mm was used as the IM length for comparison to the other estimates.

L/R-AAOCA, anomalous aortic origin of the left/right coronary artery; CTA, computed tomography angiography; LAD, left anterior descending coronary artery; MB, myocardial bridge; mm, millimeter; R, reimplantation; U, unroofing.

**Figure 1:**
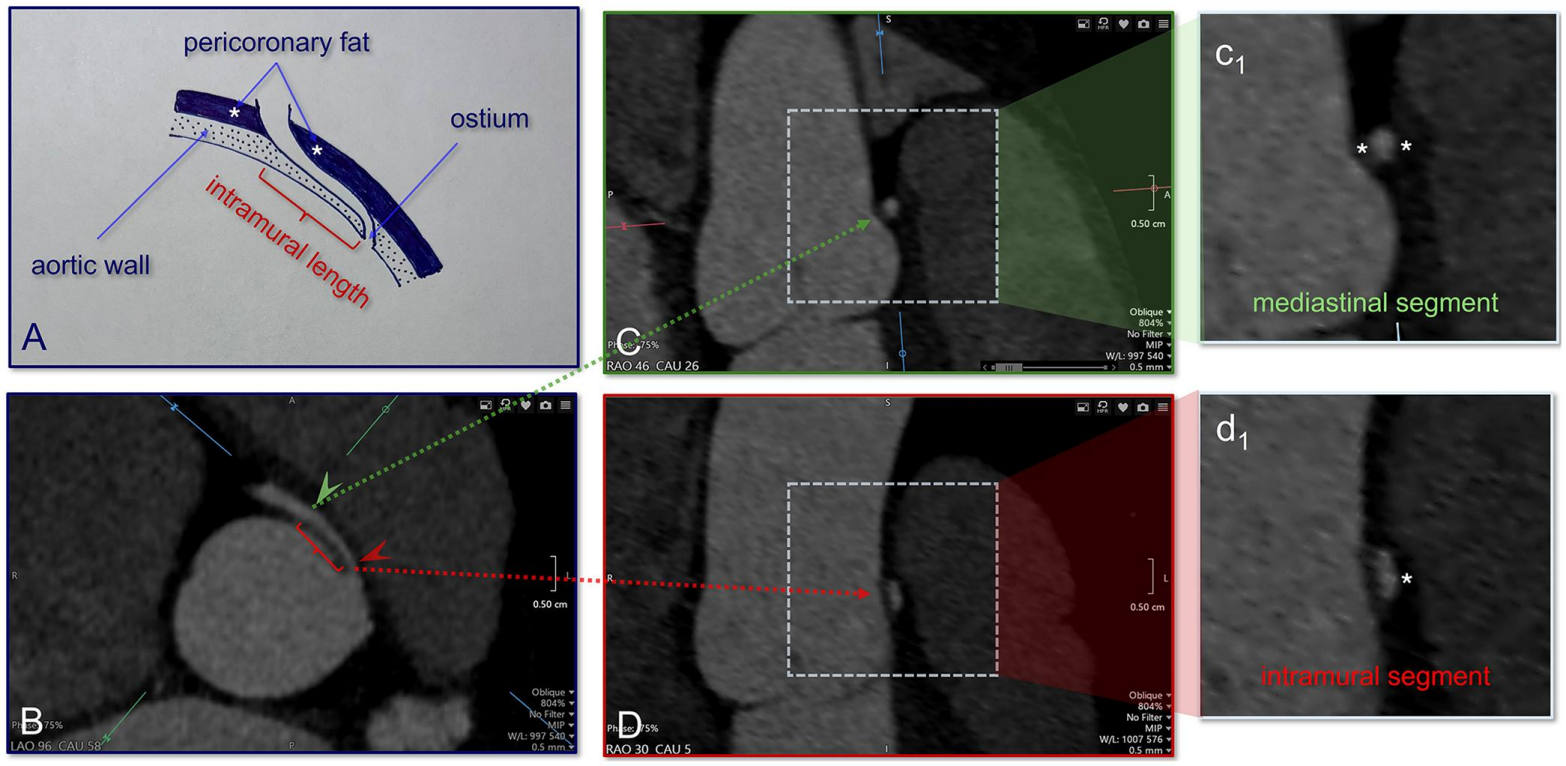
Schematic and CT demonstration of the intramural vs. mediastinal segments of the anomalous right coronary artery. **(A)** Conceptual illustration showing the relationship of the coronary artery to the aortic wall, highlighting the intramural course where the vessel is embedded within the aortic media (dotted region) and the surrounding peri-coronary fat external to the aorta. Asterisks indicate areas of peri-coronary fat (fat pad) **(B)** Axial coronary CT image identifying the proximal coronary artery and its spatial relationship to the aortic wall. **(C)** Oblique multiplanar reconstruction (green panel) focused on the mediastinal segment at the level of the green arrowhead seen on panel B, with (c_1_) showing a magnified view of the round vessel surrounded by peri-coronary fat (asterisks). **(D)** Corresponding oblique reconstruction (red panel) aligned with the intramural course at the level of the green arrowhead seen on panel B, with (d_1_) demonstrating the elliptical coronary segment embedded within the aortic wall with fat pad only on the external side of the coronary artery (asterisk). Together, these panels illustrate the imaging approach for distinguishing mediastinal from intramural coronary segments and for localizing the intramural length. CT, computed tomography.

The intramural (IM) segment, where the coronary artery travels within the aortic wall, is an important factor that influences the severity of this condition due to risk of dynamic compression with exertion (9–11). The IM length has been linked to the likelihood of adverse cardiac events in adults and has conventionally been measured from CTA images using the fat pad method or elliptical shape method (12). Accurate estimation of IM length is necessary for surgical planning to repair an anomalous coronary artery with an IM course. Studies have shown that patients with a short IM segment are more likely to benefit from transection and reimplantation of the anomalous coronary, as unroofing to place the ostium into the correct sinus would disrupt the aortic valve. In contrast, when the IM segment is longer, unroofing positions the ostium in the correct sinus while preserving aortic valve morphology and function (13–16). There is variable inter-observer agreement between radiologic estimates of IM length and surgical findings, depending on the radiologic method used (17). An example of a radiologic measurement is shown in Figure 1 along with several cross-sectional views of the anomalous coronary lumen.

To our knowledge, no studies have used computational techniques for estimating the IM length (18–21). The objective of this study is to develop such an approach and to demonstrate that it is accurate compared to surgically measured values.

## Methods

A single-institution, retrospective study was conducted of AAOCA patients who underwent surgical repair. Data was collected with approval from our institutional review board. The study cohort consisted of a consecutive patient series with intramural segment length measured by CTA. In this cohort, cases of anomalous aortic origin of the left coronary artery (L-AAOCA) had an interarterial course between the aorta and pulmonary trunk. The computational method for estimating IM length was compared against radiologic measurements. Both computational and radiologic estimates were then compared against surgical measurements, which served as the reference standard. The radiologic measurements were taken from clinical reports made by two cardiovascular radiologists at our institution. The radiologists used two previously described methods to determine intramurality: elliptical shape and peri-coronary fat (17). The intramural length was taken as the distance from the ostium of the coronary artery to the point at which the coronary artery exits the aortic wall and enters the mediastinum. The intramural segment of the coronary artery exhibits an elliptical lumen while embedded within the aortic wall, transitioning to a circular configuration upon exiting into the mediastinum. Radiologists use the elliptical shape method to measure the distance from the coronary ostium to the point at which the vessel becomes round, as a proxy for IM length (17). For the elliptic shape method, oblique coronal reconstructions were visualized at a post-processed resolution of 0.3 mm. The visual impression of the coronary artery in its proximal interarterial course, where a height-to-width ratio of greater than 1.3:1 was used as a surrogate for intramurality (although in most instances this ratio was close to 2:1), augured well for consistency. The IM length was measured from the inner lip of the anomalous vessel, as it took off from the opposite sinus, to the end point where the anomalous vessel changed to a round shape. The measured length was defined as the straight-line distance from the inner lip to this end point. The anomalous coronary artery has a fat pad in the intramural portion as opposed to a fully circumferential peri-coronary fat cuff in the mediastinal portion. Radiologists use the fat pad method to measure the distance from the ostium to where the coronary has a peri-coronary cuff to generate an intramural length (17). The peri-coronary soft tissue attenuation was determined visually with commonly followed criteria of fat vs. soft tissue attenuation. The Hounsfield units for fat were always negative (−50 to −200) and soft tissue density measured in the positive range (+50 to +200), which allowed for a clear distinction in most cases. In some patients, the gap between the anomalous vessel and aortic wall was barely perceptible, and we could not use the peri-coronary fat to help us in determining the IM length. Refer to Figure 1. Between these two methods, the predominant one used for determining IM length was the elliptical shape approach. The approach based on the peri-coronary cuff of fat was used only as a complimentary tool.

Measurements performed during surgery were taken to be the reference standard and were used in the evaluation of our approach. At our institution, intramural lengths are measured at surgery by placing a transmural suture through the aortic wall, immediately beyond the distal aspect of where the coronary artery fully emerges from the aorta. Silk string is extended from this point to the coronary ostium. This segment of string is cut, brought out of the surgical site, and placed onto a sterile ruler with millimeter resolution. The surgically measured intramural length is reported as the length of this segment of string to the nearest millimeter (14). Refer to Table 1 for intramural length measurements, ostium locations, and surgical repair types for the study cohort.

**Figure 2:**
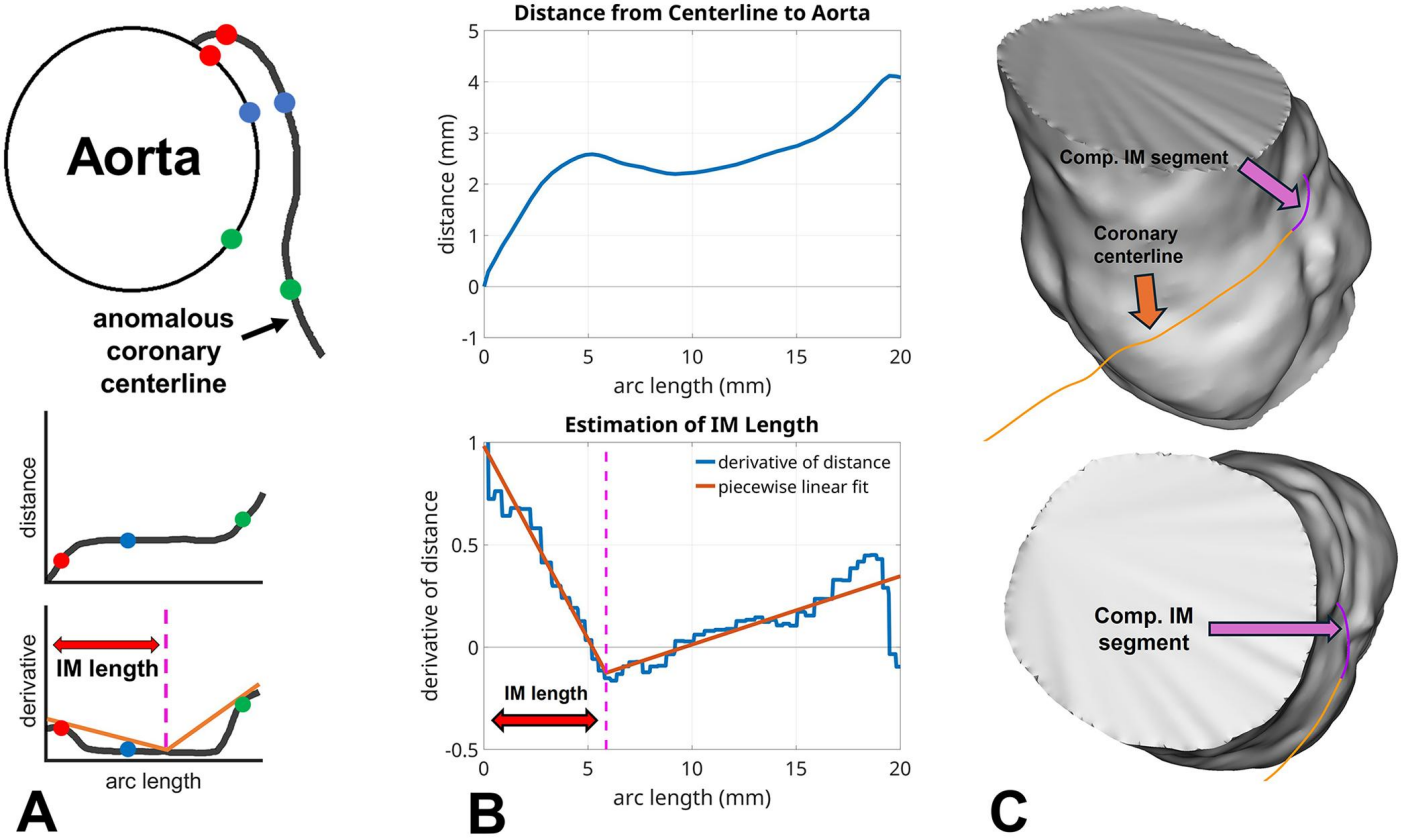
**(A)** A schematic of the aortic segmentation and coronary centerline from an axial viewpoint with idealized distance and derivative curves. Distinguished points along the segmentation and the corresponding closest points on the segmentation are marked in red, green, and blue on the schematic and the curves. **(B)** Plots of distance (top) from the coronary centerline to the aorta along the arc length of the centerline and its derivative (bottom). Arc length corresponds to the distance along the centerline starting from the coronary ostium. The piecewise linear fit to the derivative is superimposed on this plot. The magenta, dashed, vertical line indicates the transition point in the piecewise linear function that defines the computational IM length estimate. **(C)** A visualization of the mesh of aorta (grey) with anomalous coronary centerline (orange) and computationally estimated IM segment (purple). IM, intramural length; Comp, computational.

For the computational approach, CTA images were imported into 3D Slicer (Kitware) to create 3D segmentations of the aorta, extending from the sinuses to a few centimeters distal to the sinotubular junction (Figure 2C). Four out of 62 patients were excluded because poor image quality from motion degradation prevented accurate 3D segmentation. Segmentations were created semi-automatically using the grow-from-seeds function in 3D Slicer. Mild smoothing was automatically performed in 3D Slicer before exporting the segmentation as a triangulated surface. The edge lengths of the exported surface were determined automatically by 3D Slicer and were approximately 0.4 mm. The CTA images were imported into SimVascular to create a centerline of the anomalous coronary artery (Figure 2C). The centerline was generated using 15–20 control points. After it was exported from SimVascular, the centerline had a point spacing of approximately 0.4 mm. The segmentation and centerline were then imported into Matlab (Mathworks). The Euclidean distance from the aortic wall to each point along the centerline was computed (Figure 2A). This calculation was done in a greedy fashion by determining the closest vertex on the aortic segmentation to each point on the centerline. The signed distance was computed to quantify what centerline points were inside the segmentation, with a negative distance corresponding to those inside and positive corresponding to those outside. Practically, only a few points at the beginning of the centerline were inside the aortic segmentation since the initial centerline point was defined proximal to the ostium. The computed distance function was then uniformly resampled with a spacing of 0.001 mm to compute its derivative (Figure 2B). The distance function's derivative was calculated to quantify rapid changes in the distance curve that might correlate with the distal end of the intramural segment. A two-part piecewise linear function was fit to the first 20 mm of the derivative of the distance function using custom Matlab code and the built-in “fit” function (Figure 2B). The piecewise linear function took the form:

y(x)=a+bx,x<k

y(x)=a+bk+c(x−k),x≥k

The variable *x* is the arc length and the parameter *k* determines the intramural length by specifying the transition point between the two pieces of this function. The Matlab fit function calculated the parameters a,b,c,andk and was implemented with a lower bound of zero on the parameter *k*. The IM length was estimated from the transition point (magenta dashed-line in Figure 2) of the fitted piecewise linear function. This point corresponds to the intersection of the two linear segments, and our hypothesis is that it approximates the location along the centerline at which the anomalous coronary exits the aortic wall.

A single operator created the 3D aortic segmentations and coronary centerlines. These were the only steps that required manual user intervention and took about 40 minutes per patient. For the segmentation in 3D Slicer, the user manually specified initial regions for the grow-from-seeds function. For the centerline generation in SimVascular, the user manually specified control points along the anomalous coronary. Future work could involve replacing the manual generation of the segmentation and centerline with automated machine-learning-based image processing methods. Once the mesh and coronary centerline were created, intramural length estimates were generated automatically with the Matlab program, which took about 4–5 minutes to run per patient. The computation of the intramural length from this program is independent of an operator, and as such, the entire method is semi-automatic.

**Figure 3:**
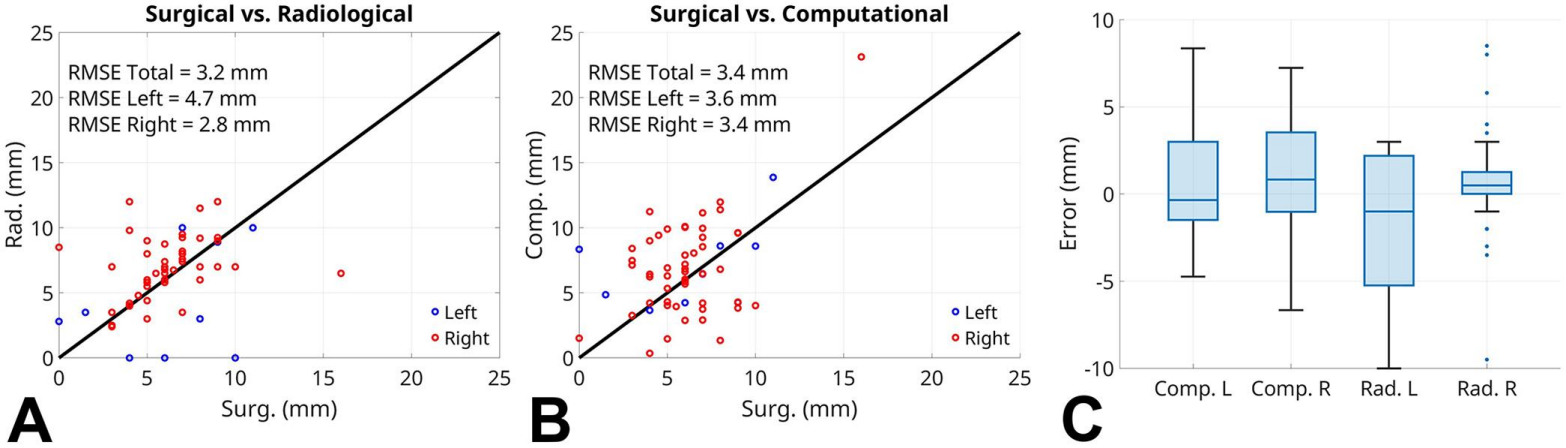
**(A)** RMSE of the radiologic method vs. surgical estimates for L- and R-AAOCA. **(B)** RMSE of computational estimates vs. surgical measurements. **(C)** Box-and-whisker plots comparing computational and radiologic errors, stratified by L- and R-AAOCA. RMSE, root-mean-square error; AAOCA, anomalous aortic origin of a coronary artery; Surg, surgical measurement; Comp, computational estimate; Rad, radiologic estimate; L, anomalous aortic origin of the left coronary artery (L-AAOCA); R, anomalous aortic origin of the right coronary artery (R-AAOCA).

The accuracy of our method was assessed using the root-mean-square error (RMSE), calculated against the surgically measured IM length. The RMSE of our computational approach was compared to the RMSE of radiologic estimates and stratified by left (L)- and right (R)-AAOCA patients. Figures 3A,B show parity plots for the radiologic and computational approaches. The Wilcoxon signed-rank test was also used to further assess similarity between the computational and radiologic approaches. Box-and-whisker plots were used to visually compare errors between the computational and radiologic methods (Figure 3C).

## Results

The study included 58 AAOCA patients: 49 R-AAOCA and 9 L-AAOCA. For the total cohort, as compared to the reference standard measurement of IM length obtained at surgery, the RMSE for the computational approach was 3.4 mm compared to 3.2 mm for the radiologic approach. For L-AAOCA patients, the RMSE was 3.6 mm for the computational approach compared to 4.7 mm for the radiologic approach. For R-AAOCA patients, the RMSE was 3.4 mm for the computational approach compared to 2.8 mm for the radiologic approach. The *p*-values for the Wilcoxon signed-rank test used to compare the computational and radiologic methods were *p* = 0.098 for the L-AAOCA cohort and *p* = 0.87 for the R-AAOCA cohort. Figure 3C displays box-and-whisker plots comparing the errors in computational and radiologic estimates, relative to the surgical measurements, which are stratified by R-AAOCA and L-AAOCA cases. In the left cohort, the computational method demonstrated a mean error of 0.7 mm (SD 3.8 mm), compared with −2.0 mm (SD 4.5 mm) for the radiologic approach. In the right cohort, the mean errors were 0.8 mm (SD 3.4 mm) for the computational method and 0.7 mm (SD 2.7 mm) for the radiologic method.

## Conclusion

The computational approach produced RMSE values comparable to the radiologic method. In addition, our technique was closer to surgical measurements, as measured by the RMSE, in the L-AAOCA cases. When viewing the box plots in Figure 3C, the outliers visible in the radiologic approach might be explained, in part, by the manual estimation required for this method. At a significance level of 5%, the Wilcoxon signed-rank test failed to reject the null hypothesis, for both the L-AAOCA and R-AAOCA cohorts, that the computational and radiologic approaches have the same median intramural length. This result provides statistical evidence for the claim that our computational method produces results that are comparable to the radiologic approach. This suggests our approach may offer a valuable supplement to the radiologic methods to inform clinical management and operative strategy. Given manually generated segmentations and centerlines, the distance function used here allowed for an automatic and objective tool for localization of the intramural segment. Future work could involve complete automation of IM length estimation if our approach can be combined with automatic methods for aortic segmentation and coronary centerline generation. Machine learning methods for image segmentation exist, but it is not clear to what extent they can be reliably applied to CTA images of AAOCA patients.

Accurate risk stratification in patients with AAOCA remains a significant challenge in clinical practice. One of the key factors influencing risk of ischemia and sudden cardiac death is the length of the intramural segment. Existing methods for measuring IM length from CTA images have demonstrated variable concordance with surgical findings (17). This study developed and tested a computational method to estimate IM length from CTA images. Our method was accurate compared to surgical measurements.

This study has limitations. The model was developed and tested in a single-institution cohort, which may limit its generalizability. The small sample size and the limited number of patients with L-AAOCA reduce the study's statistical power. Further validation is needed to confirm that different operators can generate comparable 3D aortic segmentations and coronary centerlines without significant variation in the computed IM length estimates. Another limitation is our choice of the surgical measurement as the reference standard. While the surgical measurement is our only available value for comparison in this pediatric cohort, it must be noted that intravascular ultrasound (IVUS) is superior as a gold-standard measurement of intramural length given that it depicts the vessel in its natural, filled state and provides sub-millimeter precision (22). The feasibility of automated, validated IVUS analysis for coronary anomalies has previously been demonstrated (23).

Additional anatomic features of AAOCA—such as acute take-off angle, slit-like ostium, minimal lumen area, and maximal luminal narrowing—have been shown to influence ischemic risk (24, 25). Incorporating these parameters, along with intramural length, into computational models may enhance risk assessment and surgical planning. Future studies involving multicenter cohorts, larger sample sizes (including more patients with the less common left AAOCA subtype), and integration into clinical workflows are warranted to evaluate whether this approach improves risk stratification, surgical planning, and patient management.

## Data Availability

The original contributions presented in the study are included in the article/Supplementary Material. Code used in this study is available in the GitHub repository https://github.com/vikramshah-01/AAOCA_IML_Estimator.git and further inquiries can be directed to the corresponding author.

## References

[1] Basso C Maron BJ Corrado D Thiene G. Clinical profile of congenital coronary artery anomalies with origin from the wrong aortic sinus leading to sudden death in young competitive athletes. J Am Coll Cardiol. (2000) 35(6):1493–501. 10.1016/S0735-1097(00)00566-0 10807452

[2] Petek BJ Churchill TW Moulson N Kliethermes SA Baggish AL Drezner JA Sudden cardiac death in National Collegiate Athletic Association athletes: a 20-year study. Circulation. (2024) 149(2):80–90. 10.1161/CIRCULATIONAHA.123.065908 37955565 PMC10843024

[3] Finocchiaro G Westaby J Sheppard MN Papadakis M Sharma S. Sudden cardiac death in young athletes: JACC state-of-the-art review. J Am Coll Cardiol. (2024) 83(2):350–70. 10.1016/j.jacc.2023.10.032 38199713

[4] Mainwaring RD Reddy VM Reinhartz O Petrossian E Punn R Hanley FL. Surgical repair of anomalous aortic origin of a coronary artery. Eur J Cardiothorac Surg. (2014) 46(1):20–6. 10.1093/ejcts/ezt614 24431169

[5] Maron BJ Doerer JJ Haas TS Tierney DM Mueller FO. Sudden deaths in young competitive athletes: analysis of 1866 deaths in the United Satates, 1980–2006. Circulation. (2009) 119(8):1085–92. 10.1161/CIRCULATIONAHA.108.804617 19221222

[6] Kim JH Baggish AL Levine BD Ackerman MJ Day SM Dineen EH Clinical considerations for competitive sports participation for athletes with cardiovascular abnormalities: a scientific statement from the American Heart Association and American College of Cardiology. J Am Coll Cardiol. (2025) 151(11):e716–61. 10.1016/j.jacc.2024.12.025 39973614

[7] Finocchiaro G Behr ER Tanzarella G Papadakis M Malhotra A Dhutia H Anomalous coronary artery origin and sudden cardiac death: clinical and pathological insights from a national pathology registry. JACC Clin Electrophysiol. (2019) 5(4):516–22. 10.1016/j.jacep.2018.11.015 31000108

[8] Cheezum MK Liberthson RR Shah NR Villines TC O’Gara PT Landzberg MJ Anomalous aortic origin of a coronary artery from the inappropriate sinus of valsalva. J Am Coll Cardiol. (2017) 69(12):1592–608. 10.1016/j.jacc.2017.01.031 28335843

[9] Doan TT Sachdeva S Bonilla-Ramirez C Reaves-O’Neal D Masand P Krishnamurthy R Anomalous aortic origin of coronary arteries in children: postoperative high-risk anatomic features. Ann Thorac Surg. (2023) 115(4):991–8. 10.1016/j.athoracsur.2022.11.024 36470562

[10] Doan TT Wilkes JK Reaves-O’Neal DL Bonilla-Ramirez C Sachdeva S Masand P Clinical presentation and medium-term outcomes of children with anomalous aortic origin of the left coronary artery: high-risk features beyond interarterial course. Circ Cardiovasc Interv. (2023) 16(5):e012635. 10.1161/CIRCINTERVENTIONS.122.012635 37192311

[11] Doan TT Sachdeva S Bonilla-Ramirez C Reaves-O’Neal DL Masand P Mery CM Ischemia in anomalous aortic origin of a right coronary artery: large pediatric cohort medium-term outcomes. Circ Cardiovasc Interv. (2023) 16(4):e012631. 10.1161/CIRCINTERVENTIONS.122.012631 37071720

[12] Gaudino M Di Franco A Arbustini E Bacha E Bates ER Cameron DE Management of adults with anomalous aortic origin of the coronary arteries: state-of-the-art review. J Am Coll Cardiol. (2023) 82(21):2034–53. 10.1016/j.jacc.2023.08.012 37855757

[13] Bonilla-Ramirez C Molossi S Sachdeva S Reaves-O’Neal D Masand P Mery CM Outcomes in anomalous aortic origin of a coronary artery after surgical reimplantation. J Thorac Cardiovasc Surg. (2021) 162(4):1191–9. 10.1016/j.jtcvs.2020.12.100 33541731

[14] Mery CM Beckerman Z. What is the optimal surgical technique for anomalous aortic origin of a coronary artery? Semin Thorac Cardiovasc Surg Ann. (2025) 28:94–100. 10.1053/j.pcsu.2025.02.006 40382131

[15] Padalino MA Jegatheeswaran A Blitzer D Ricciardi G Guariento A. Surgery for anomalous aortic origin of coronary arteries: technical safeguards and pitfalls. Front Cardiovasc Med. (2021) 8:626108. 10.3389/fcvm.2021.626108 34055925 PMC8149602

[16] Brothers JA Frommelt MA Jaquiss R Myerburg RJ Fraser CD Tweddell JS. Expert consensus guidelines: anomalous aortic origin of a coronary artery. J Thorac Cardiovasc Surg. (2017) 153(6):1440–57. 10.1016/j.jtcvs.2016.06.066 28274557

[17] Krishnamurthy R Masand PM Jadhav SP Molossi S Zhang W Agrawal HM Accuracy of computed tomography angiography and structured reporting of high-risk morphology in anomalous aortic origin of coronary artery: comparison with surgery. Pediatr Radiol. (2021) 51(8):1299–310. 10.1007/s00247-021-05011-0 33755749

[18] Lo Rito M Romarowski RM Rosato A Pica S Secchi F Giamberti A Anomalous aortic origin of coronary artery biomechanical modeling: toward clinical application. J Thorac Cardiovasc Surg. (2020) S0022-5223(20):32430–2. 10.1016/j.jtcvs.2020.06.150 32950237

[19] Jiang MX Khan MO Ghobrial J Rogers IS Pettersson GB Blackstone EH Patient-specific fluid-structure simulations of anomalous aortic origin of right coronary arteries. JTCVS Tech. (2022) 13:144–62. 10.1016/j.xjtc.2022.02.022 35711199 PMC9196314

[20] Stark AW Giannopoulos AA Pugachev A Shiri I Haeberlin A Räber L Application of patient-specific computational fluid dynamics in anomalous aortic origin of coronary artery: a systematic review. J Cardiovasc Dev Dis. (2023) 10(9):384. 10.3390/jcdd10090384 37754814 PMC10532130

[21] Shiri I Baj G Mohammadi Kazaj P Bigler MR Stark AW Valenzuela W AI-based detection and classification of anomalous aortic origin of coronary arteries using coronary CT angiography images. Nat Commun. (2025) 16:3095. 10.1038/s41467-025-58362-9 40169568 PMC11961624

[22] Stark AW Bigler MR Räber L Gräni C. True pulsatile lumen visualization in coronary artery anomalies using controlled transducer pullback and automated IVUS segmentation. JACC Case Rep. (2025) 30(22):104741. 10.1016/j.jaccas.2025.104741 40780785 PMC12426529

[23] Stark AW Kazaj PM Balzer S Ilic M Bergamin M Kakizaki R Automated intravascular ultrasound image processing and quantification of coronary artery anomalies: the AIVUS-CAA software. Comp Methods Programs Biomed. (2025) 272:109065. 10.1016/j.cmpb.2025.109065 40972478

[24] Stark AW Matthey-de-l’Endroit RL Ferroni A Kakizaki R Bigler MR Biccirè FG Coronary CT anatomy-based prediction of invasively assessed hemodynamic significance in middle-aged patients with right coronary artery anomaly: the NARCO study. Circulation. (2025) 151(8):578–80. 10.1161/CIRCULATIONAHA.124.071637 39993033

[25] Bigler MR Ashraf A Seiler C Praz F Ueki Y Windecker S Hemodynamic relevance of anomalous coronary arteries originating from the opposite sinus of valsalva-in search of the evidence. Front Cardiovasc Med. (2021) 7:591326. 10.3389/fcvm.2020.591326 33553251 PMC7859106

